# Low Plasma Levels of Adiponectin Do Not Explain Acute Respiratory Distress Syndrome Risk: a Prospective Cohort Study of Patients with Severe Sepsis

**DOI:** 10.1186/s13054-016-1244-2

**Published:** 2016-03-16

**Authors:** Jessica A. Palakshappa, Brian J. Anderson, John P. Reilly, Michael G. S. Shashaty, Ryo Ueno, Qufei Wu, Caroline A. G. Ittner, Anna Tommasini, Thomas G. Dunn, Dudley Charles, Altaf Kazi, Jason D. Christie, Nuala J. Meyer

**Affiliations:** Division of Pulmonary, Allergy, and Critical Care, Perelman School of Medicine, University of Pennsylvania, Hospital of the University of Pennsylvania, 3400 Spruce Street, Philadelphia, PA 19104 USA; Center for Clinical Epidemiology and Biostatistics, Perelman School of Medicine, University of Pennsylvania, Blockley Hall, 423 Guardian Drive, Philadelphia, PA 19104 USA; Graduate School of Medicine, The University of Tokyo, 7-3-1, Hongo, Bunkyo-ku, Tokyo, 1130033 Japan

**Keywords:** Acute respiratory distress syndrome, Adiponectin, Sepsis, Obesity

## Abstract

**Background:**

Obesity is associated with the development of acute respiratory distress syndrome (ARDS) in at-risk patients. Low plasma levels of adiponectin, a circulating hormone-like molecule, have been implicated as a possible mechanism for this association. The objective of this study was to determine the association of plasma adiponectin level at ICU admission with ARDS and 30-day mortality in patients with severe sepsis and septic shock.

**Methods:**

This is a prospective cohort study of patients admitted to the medical ICU at the Hospital of the University of Pennsylvania. Plasma adiponectin was measured at the time of ICU admission. ARDS was defined by Berlin criteria. Multivariable logistic regression was used to determine the association of plasma adiponectin with the development of ARDS and mortality at 30 days.

**Results:**

The study included 164 patients. The incidence of ARDS within 5 days of admission was 45 %. The median initial plasma adiponectin level was 7.62 mcg/ml (IQR: 3.87, 14.90) in those without ARDS compared to 8.93 mcg/ml (IQR: 4.60, 18.85) in those developing ARDS. The adjusted odds ratio for ARDS associated with each 5 mcg increase in adiponectin was 1.12 (95 % CI 1.01, 1.25), p-value 0.025). A total of 82 patients (51 %) of the cohort died within 30 days of ICU admission. There was a statistically significant association between adiponectin and mortality in the unadjusted model (OR 1.11, 95 % CI 1.00, 1.23, p-value 0.04) that was no longer significant after adjusting for potential confounders.

**Conclusions:**

In this study, low levels of adiponectin were not associated with an increased risk of ARDS in patients with severe sepsis and septic shock. This argues against low levels of adiponectin as a mechanism explaining the association of obesity with ARDS. At present, it is unclear whether circulating adiponectin is involved in the pathogenesis of ARDS or simply represents an epiphenomenon of other unknown functions of adipose tissue or metabolic alterations in sepsis.

## Background

Acute respiratory distress syndrome (ARDS) is a significant complication of severe sepsis, with a mortality rate approaching 50 % [1]. Body composition and metabolic factors have been shown to modify the risk of developing ARDS in at-risk patients. For example, observational studies have demonstrated association between diabetes and decreased risk of ARDS [2–5] and between obesity and increased risk of ARDS [6]. Despite the increased risk of developing ARDS, obese patients do not appear to have an increased risk of death from ARDS and may, in fact, have reduced mortality compared to non-obese patients [7–10]. The mechanisms underlying why the incidence of ARDS may be higher yet survival better in obese patients, one example of the ‘obesity paradox’, are incompletely understood [11, 12]. One proposed explanation is that obesity itself primes the lung for the development of lung injury [13].

Obese patients have alterations in the release and response of adipokines, circulating hormone-like molecules released from adipose tissue. Recent preclinical studies have focused on the role of adipokines in the mechanisms underlying obesity-associated ARDS. Adiponectin, one such adipokine, has anti-inflammatory properties [14]. Plasma levels of adiponectin are reduced in obese compared to lean individuals [15]. Adiponectin may modulate lung inflammation through multiple pathways. Adiponectin protects against systemic inflammation by promoting the clearance of apoptotic cells by macrophages [16]. In addition, adiponectin receptors are expressed on alveolar macrophages and pulmonary endothelial cells though, at present, the role of adiponectin in lung homeostasis has not been fully elucidated [13, 17–20]. Animal studies have suggested that adiponectin may play a lung-protective role in the setting of infection [21, 22], leading to the hypothesis that low levels of adiponectin in obese individuals may predispose the lung to an injury pattern, potentiating ARDS.

Although exogenous administration of adiponectin is protective against lung injury in animal models, the relationship between plasma adiponectin and lung injury in human patients is less clear. Prior observations have reported that higher levels of plasma adiponectin are associated with increased mortality in patients with respiratory failure but are not associated with severity of lung disease [23, 24]. The primary objective of this study was to determine the association between plasma adiponectin level at admission to the intensive care unit (ICU) with the development of ARDS in patients with severe sepsis and septic shock. Based on preclinical studies, we hypothesized that low adiponectin levels at the time of ICU admission would be associated with ARDS in at-risk patients. In addition, we sought to determine the relationship between adiponectin and mortality in septic patients.

## Methods

### Study design

The Molecular Epidemiology of Severe Sepsis in the ICU (MESSI) study is a prospective cohort study of sepsis patients admitted to the medical ICU at the Hospital of the University of Pennsylvania, an urban tertiary referral center. For this study, we included 164 consecutive patients enrolled in the MESSI cohort from 6 January 2011 through 15 March 2012, for whom plasma samples were available for analysis.

### Study population

The MESSI cohort has been described previously [25, 26]. Patients are eligible for enrollment in the MESSI cohort if the primary reason for ICU admission is severe sepsis as defined by the American College of Chest Physicians consensus definition [27]. The consensus criteria include (1) two or more systemic inflammatory response syndrome criteria, (2) known or strongly suspected infection, and (3) evidence of organ dysfunction or shock. Exclusion criteria were lack of commitment to life-sustaining measures at enrollment or admission from a long-term acute care facility. For this study, we also excluded patients admitted to the ICU from an outside hospital to ensure all plasma samples were from ICU admission. The Institutional Review Board at the University of Pennsylvania approved the study, Protocol 808542. This study was approved with a waiver of timely consent. Consent was obtained from patients or surrogates as soon as feasible.

### Data collection

Trained research personnel collected clinical data prospectively, including baseline demographics and chronic health information. Physiologic information and laboratory values were collected daily over the first 6 days of the subject’s ICU stay. Height and weight were extracted from patient interview or the electronic medical record to determine body mass index (BMI) at the time of ICU admission. BMI categories were defined by the World Health Organization (WHO) categories: BMI ≥30 kg/m^2^ was considered obese [28]. All plasma samples were obtained from the day of ICU admission. The mean duration between plasma sample and time when patients met criteria for ARDS was 12.29 hours (range of 127.36 hours before to 21.66 hours after ARDS criteria were met). Adiponectin was measured in duplicate using a human adiponectin enzyme-linked immunosorbent assay (R&D Systems, Minneapolis, MN).

### Outcome definition

The primary outcome was the development of ARDS within 5 days of ICU admission. ARDS was defined by Berlin criteria: acute-onset bilateral infiltrates on chest radiograph consistent with pulmonary edema and not fully explained by cardiac failure with arterial partial pressure of oxygen/inspired oxygen fraction (P_a_O_2_/FiO_2_) ≤300 [29]. Chest radiographs were independently evaluated by two physician-investigators blinded to other data, with adjudication as necessary as previously described [30]. To be defined as having ARDS, a subject had to meet the Berlin criteria for both radiographs and arterial blood gas within a 24-hour period [25, 29]. Mortality at 30 days was determined using data available in the electronic health record. If there was no healthcare encounter within 30 days of ICU admission, we performed an online obituary search. Online obituary search was used to determine the status of four patients (2 %) in the cohort whose vital status was ambiguous in the medical record.

### Statistical analysis

#### Plasma adiponectin and ARDS

Wilcoxon rank sum and chi square tests were performed to evaluate differences in baseline characteristics between patients with ARDS and patients without ARDS. We used Student’s *t* test of log-transformed adiponectin to compare adiponectin levels in patients with and without ARDS. Multivariable logistic regression was used to determine the association between plasma adiponectin on the day of admission and ARDS within 5 days of ICU admission, adjusting for potential confounders. We created two multivariable models. In the first model, we decided *a priori* to adjust for the following covariates, given their known association with serum adiponectin levels: history of chronic liver disease [31, 32], diabetes [33], and BMI [34]. BMI was included as a continuous variable in all analyses. We also included potential confounders that were associated with adiponectin or ARDS in bivariate analysis (*p* value <0.25) [35]. We evaluated effect modification of the association between adiponectin and ARDS by BMI using the likelihood ratio test. We used post-estimation marginal analysis of final logistic regression models to determine the standardized risk of ARDS at several percentiles of plasma adiponectin [36]. In a second multivariable model, we determined the association of plasma adiponectin with ARDS after adjusting for BMI, pulmonary source of infection, history of diabetes, and Acute Physiology and Chronic Health Evaluation (APACHE) III. We also performed a subsequent unadjusted analysis excluding patients who developed ARDS in the first 6 hours after the plasma sample was obtained.

#### Plasma adiponectin and mortality

We used multivariable logistic regression to test the association between plasma adiponectin on the day of ICU admission and 30-day mortality, including ARDS as a covariate. We also created a multivariable model adjusting for ARDS, history of chronic liver disease, diabetes, and BMI. Further confounders were considered in the final logistic regression models if they were associated with adiponectin or mortality in bivariate analysis (*p* value <0.25) [35]. We evaluated effect modification of the association between adiponectin and 30-day mortality by BMI using the likelihood ratio test. Mortality status was unknown in three patients. All statistical analyses were performed with Stata/IC 13.0 (StataCorp LP, College Station, TX).

## Results

The study sample included 164 patients with severe sepsis or septic shock who required ICU admission. The incidence of ARDS within 5 days of ICU admission was 45 %. Among those patients who developed ARDS, the prevalence of ARDS on the day of ICU admission was 67 % and a total of 88 % had ARDS by ICU day 2. Characteristics of study participants are presented in Table 1. Patients who developed ARDS had a higher median BMI compared to those did not, but this was not statistically significant. The incidence of ARDS was lower in diabetic patients, consistent with prior studies, though this was not statistically significant [2–4]. Those who developed ARDS were more likely to have a history of liver disease, shock on the day of admission, and a pulmonary source of sepsis. Patients with ARDS also had increased severity of illness and higher 30-day mortality compared to those patients who did not develop ARDS. Patients who died were more likely to be of white race, immunocompromised, and have a history of liver disease (Table 2). ARDS was a significant risk factor for mortality in this cohort.

**Table 1:** Patient characteristics by ARDS status

	Non-ARDS (n = 90)	ARDS (n = 73)	*P*-value
Age (median, IQR)	63 (55, 72)	58 (52, 68)	0.09
Female sex (n, %)	35 (39)	31 (42)	0.64
White race (n, %)	48 (53)	41 (56)	0.72
Tobacco use (n, %)			0.81
Never smoker	24 (27)	22 (30)	
Ever smoker	54 (60)	39 (53)	
Current smoker	9 (10)	8 (11)	
Unknown	3 (3)	4 (5)	
BMI (median, IQR)	23.6 (20.3, 28.6)	25.6 (21.3, 31.0)	0.15
Weight class (n, %)			0.14
BMI ≤18.5 kg/m^2^	11 (12)	8 (11)	
BMI 18.5–25 kg/m^2^	43 (48)	23 (32)	
BMI 25–30 kg/m^2^	18 (20)	19 (26)	
BMI ≥30 kg/m^2^	18 (20)	23 (32)	
History of diabetes (n, %)	37 (41)	21 (28)	0.10
History of chronic liver disease (n, %)	4 (4)	14 (19)	0.003
History of chronic kidney disease (n, %)	9 (10)	9 (12)	0.66
Immunocompromised (n, %)	44(49)	38 (52)	0.69
History of organ transplantation (n, %)	8 (9)	4 (6)	0.42
Pulmonary source of sepsis (n, %)	25 (28)	38 (52)	0.002
Shock on Admission (n, %)	51 (57)	54 (74)	0.03
APACHE III (median, IQR)	72 (61, 90)	85 (73, 98)	0.003
30-day mortality (n, %)	28 (32)	54 (74)	<0.001

Values obtained using Wilcoxon ranksum and chi square tests. Abbreviations: *ARDS* = Acute Respiratory Distress Syndrome; *BMI* = Body Mass Index; *APACHE* = Acute Physiology, Age, Chronic Health Evaluation

**Table 2:** Patient characteristics by 30-day mortality

	Alive at 30 days (n = 79)	Died at 30 days (n = 82)	*P* value
Age, median (IQR)	61 (54, 71)	63 (53, 70)	0.82
Female sex, n (%)	34 (43)	31 (38)	0.50
White race, n (%)	33 (42)	54 (66)	0.002
Body mass index, median (IQR)	25.0 (21.6, 30.6)	24.5 (20.7, 30.2)	0.42
History of diabetes, n (%)	32 (41)	24 (29)	0.13
History of chronic liver disease, n (%)	3 (4)	15 (18)	0.004
History of chronic kidney disease, n (%)	8 (10)	10 (12)	0.66
Immunocompromised, n (%)	33 (42)	50 (61)	0.02
History of organ transplant, n (%)	6 (8)	6 (7)	0.96
Pulmonary source of sepsis, n (%)	27 (34)	36 (44)	0.21
Shock on admission, n (%)	47 (59)	57 (68)	0.25
ARDS, n (%)	19 (24)	54 (66)	<0.001

Values obtained using Wilcoxon rank sum and chi square tests. *ARDS* acute respiratory distress syndrome

The median initial plasma adiponectin level was 7.62 mcg/ml (IQR 3.87, 14.90) in those without ARDS compared to 8.93 mcg/ml (IQR 4.60, 18.85) in those who developed ARDS (*p* value 0.03). Consistent with prior studies, adiponectin level was inversely associated with BMI (Spearman *ρ* –0.2, *p* value = 0.01). The median adiponectin concentration was 7.08 mcg/ml (IQR 2.88, 11.43) in the obese and 8.84 mcg/ml (IQR 4.56, 18.53) in the non-obese. Adiponectin was also weakly correlated with APACHE III score (Spearman *ρ* 0.19, *p* value 0.02).

**Fig. 1:**
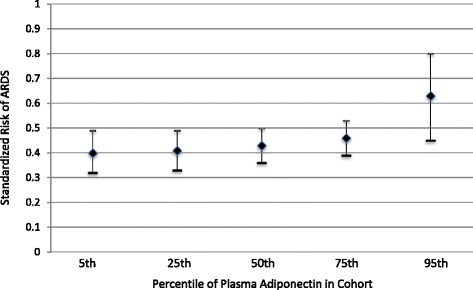
Standardized risk of acute respiratory distress syndrome (*ARDS*) by percentile of plasma adiponectin in cohort

The adjusted odds ratio (OR) for ARDS associated with each 5 mcg increase in adiponectin was 1.12 (95 % CI 1.01, 1.25, *p* value 0.025) in a model that included BMI, history of diabetes, pulmonary source of sepsis, and APACHE III (Table 3). The adjusted OR for ARDS associated with each 5 mcg increase in adiponectin was 1.11 (95 % CI 1.00, 1.23, *p* value 0.048) in a multivariable model including age, BMI, diabetes, history of chronic liver disease, pulmonary source of infection, and shock at the time of admission (Table 3). The standardized risk of ARDS holding constant all factors in the adjusted model was 40 % at an adiponectin plasma level of 1.49 mcg/ml, representing the 5^th^ percentile of plasma adiponectin. This risk increased to 62 % at an adiponectin level of 56.55 mcg/ml, representing the 95^th^ percentile of plasma adiponectin in the cohort (Fig. 1). There was no evidence of effect modification by BMI on the association between adiponectin and ARDS (*p* value for interaction term 0.50).

**Table 3:** Multivariable analyses of association of adiponectin levels at admission with ARDS

	Odds ratio per 5 mcg/ml increase in adiponectin (95 % CI)	*P* value
Unadjusted	1.12 (1.01, 1.23)	0.027
Multivariable model 1^a^	1.12 (1.01, 1.25)	0.025
Multivariable model 2^b^	1.11 (1.00, 1.23)	0.048

^a^Adjusted for diabetes, body mass index (BMI), pulmonary source of sepsis, Acute Physiology and Chronic Health Evaluation III. ^b^Adjusted for age, diabetes, BMI, history of liver disease, pulmonary source of sepsis, and shock at time of admission. *ARDS* acute respiratory distress syndrome

In a sensitivity analysis, we examined the association between plasma adiponectin and ARDS after excluding patients who met the criteria for ARDS within the first 6 hours after the plasma sample was obtained [37]. We were missing the time stamp for one plasma sample and this patient was excluded from this analysis. A total of 90 patients did not develop ARDS, 41 patients met the criteria for ARDS within 6 hours of admission, and 31 met the criteria for ARDS at least 6 hours after their initial plasma sample was obtained. After excluding the 41 patients who developed ARDS within the first 6 hours, the unadjusted OR for ARDS associated with each 5 mcg increase in adiponectin was 1.13 (95 % CI 1.00, 1.28 *p* value 0.056).

A total of 82 patients (51 %) from the cohort died within 30 days of ICU admission. There was a statistically significant association between adiponectin and mortality in the unadjusted model (OR 1.11, 95 % CI 1.00, 1.23, *p* value 0.04). In a model adjusted for ARDS, the association between adiponectin and mortality was attenuated (OR 1.08, 95 % CI 0.96, 1.21, *p* value 0.19) (Table 4). In the complete multivariable model adjusting for BMI, race, history of diabetes, history of liver disease, immunocompromised status, pulmonary source of infection, and ARDS, the association was further attenuated (OR 1.02, 95 % CI 0.92, 1.15, *p* value 0.66) (Table 4). There was no effect modification by BMI (*p* value for interaction term 0.29).

**Table 4:** Multivariable analyses of association between adiponectin levels at admission and mortality

	Odds ratio per 5mcg/ml increase in adiponectin (95 % CI)	*P* value
Unadjusted	1.11 (1.00, 1.23)	0.04
Adjusted for ARDS	1.08 (0.96, 1.21)	0.19
Multivariable model^a^	1.02 (0.92, 1.15)	0.66

^a^Adjusted for history of liver disease, diabetes, body mass index, race, immunocompromised status, pulmonary source of infection, and acute respiratory distress syndrome (ARDS)

## Discussion

In this study, we found that low plasma levels of adiponectin at the time of admission to the intensive care unit were not associated with the development of ARDS in patients with severe sepsis or septic shock. In contrast, patients with ARDS on admission, or who later developed ARDS, demonstrated higher plasma adiponectin levels than those patients without ARDS. In addition, we found high plasma adiponectin was associated with 30-day mortality in bivariate analysis; this association was no longer significant in a multivariable model including ARDS.

Adiponectin is a highly abundant circulating hormone with pleotropic effects on numerous endocrine functions and cell types. Plasma levels of adiponectin are reduced in obese individuals likely due to selective suppression of its synthesis in adipocytes [15]. Animal studies have suggested that adiponectin may play a lung protective role in the setting of sepsis. For example, adiponectin-deficient mice were found to have profoundly reduced survival in response to cecal ligation and puncture [21] and adiponectin administration was shown to attenuate lung injury in an endotoxin (lipopolysacchararide, LPS) animal model [22]. Shah et al. studied three distinct animal models of hypoadiponectinemia and found all three models were more susceptible to developing lung injury from LPS when compared with wild-type/lean animals [38]. Repletion of adiponectin protected against the development of this lung injury, leading the authors to suggest that low serum adiponectin levels should be tested as a marker for identifying individuals at risk of developing ARDS [38].

Whereas animal data implicate low adiponectin states as potentiating lung injury, our study and others in human populations do not endorse an association between low circulating adiponectin and adverse outcomes. In a population of critically ill patients requiring mechanical ventilation, 21 % of whom had ARDS, high serum adiponectin levels at admission were associated with increased 28-day mortality [24]. In a subsequent analysis of plasma samples from over 800 patients with established ARDS, there was no relationship between baseline adiponectin level and severity of illness, severity of lung injury, or mortality, though higher adiponectin levels were associated with mortality among patients with non-pulmonary but not direct pulmonary ARDS [23]. Finally, Ahasic and colleagues examined the association between genetic variations in adiponectin genes and survival in ARDS [39]. These authors observed increased mortality among patients with ARDS in homozygotes for the rs2082940 variant of adiponectin. Plasma adiponectin levels were not reported for Ahasic’s study, however this variant has been previously shown to be associated with increased ambulatory levels of adiponectin [40].

To our knowledge, ours is the first study to examine plasma adiponectin levels with the development of ARDS in patients admitted to the ICU with severe sepsis or septic shock. Our finding that low plasma adiponectin levels are not associated with ARDS is in contrast to results from models of LPS-induced lung injury in obesity-prone mice, where exogenous adiponectin upregulated vascular barrier-enhancing molecules and attenuated lung injury [38]. Our results argue against low circulating adiponectin as a mediator of the observed increase in ARDS risk faced by obese patients. Furthermore, our findings argue against low adiponectin level at the time of admission as a marker for identifying patients at increased risk of developing ARDS. Whereas high levels of adiponectin in ambulatory patients are associated with vascular protection and reduced risk of myocardial infarction [41], the significance of very high levels during critical illness are unknown. Our group and others have demonstrated differential gene and plasma regulation of inflammation during resting and evoked states [42–45] and it may be that adiponectin has pleiotropic effects in different contexts. Alternatively, it may be that high circulating levels of adiponectin reflect some level of stress detected by adipocytes without implicating a causal role for adiponectin in the development of ARDS or for mortality in ARDS. Future mechanistic studies will be necessary to delineate whether sepsis-evoked adiponectin response serves vascular barrier-enhancing functions in this context.

We found high plasma adiponectin was associated with 30-day mortality in bivariate analysis consistent with prior studies that have also found an association between high adiponectin levels and increased mortality. Koch et al. found higher baseline adiponectin levels were associated with increased ICU mortality in a cohort of patients with sepsis, independent of BMI [46]. Similarly, Walkey reported that high adiponectin levels early in respiratory failure are associated with mortality, with no effect modification by BMI [24]. In our cohort of patients with severe sepsis and septic shock, the association of high adiponectin with mortality was no longer significant after adjusting for ARDS. In the context of these prior studies, it is possible that the reason we did not find an association in the multivariable model is that ARDS is on the causal pathway for the association between plasma adiponectin level and mortality.

There are several limitations to our study. This cohort comprised patients from a single academic center that serves as a large referral center for oncology and transplant patients. As such, our cohort includes a high proportion of immunocompromised patients, which may limit the generalizability of our findings. Misclassification of BMI class is possible in our cohort. BMI is an imperfect measure in the ICU where weight often reflects the acute illness or fluid resuscitation, and height may be erroneously recorded in the supine patient. We used weight measurements collected at the time of admission to minimize the effects of critical illness and fluid resuscitation on this measurement. In addition, we were unable to control for glucose intake or insulin administration immediately prior to plasma analysis. Given our samples are drawn from the time of admission, we do not expect this to have a significant impact on our results. We only measured one adipokine in this study and did not perform fractionation studies. Adiponectin circulates in three main forms: trimers, hexamers, and high-molecular weight multimers [14]. Future studies could examine the association between different sub-fractions of adiponectin and ARDS. Further, the relationship between adiponectin and other biomarkers of endothelial activation, such as von Willebrand factor and angiopoietin-2, may be important to explore [47, 48].

The possibility of reverse causality must be considered in interpreting our results. We do not have plasma samples available for this cohort prior to the onset of sepsis and it is possible these patients initially had low levels of plasma adiponectin that increased in response to sepsis. However, after excluding patients who met the criteria for ARDS in the first 6 hours after the plasma sample was obtained, the association between high plasma adiponectin levels and ARDS demonstrated a similar effect size. While we cannot implicate a causal role for adiponectin in the pathogenesis of ARDS from these results, further mechanistic studies should be performed to understand the role of adiponectin in the development of lung injury.

This study has several strengths. This was a well-characterized and diverse cohort of septic patients, with careful outcome ascertainment. The ARDS phenotype was determined by multiple-physician consensus review. Prior studies examining the association between plasma adiponectin and patient outcomes have included plasma samples from patients previously enrolled in randomized controlled trials [23, 24]. Our study adds to this literature in several important ways. First, this cohort includes a diverse group of patients with sepsis allowing us to examine the association between plasma adiponectin and the development of ARDS in addition to the outcome of mortality. In addition, our study is an observational cohort study and includes many patients who typically would not be enrolled in randomized controlled trials and may have important differences compared to the enrolled critically ill trial population [49]. Finally, plasma samples were available from the time of admission to the ICU in our cohort, allowing us to examine plasma adiponectin levels at a time when screening for at-risk patients would be feasible and at a time point earlier than plasma sample analysis in randomized controlled trials. In the ARDS Network Fluid and Catheter Treatment Trial (FACTT), the source of plasma samples for the study by Walkey et al. that examined the association of adiponectin with patient outcomes, the mean time between admission to the ICU and first protocol instruction was approximately 42 hours [50].

## Conclusions

We found that high plasma levels of adiponectin are associated with ARDS in patients with severe sepsis and septic shock. This association was independent of BMI. At present, it is unclear whether circulating adiponectin is involved in the pathogenesis of ARDS or simply represents an epiphenomenon of other unknown functions of adipose tissue or metabolic alterations in sepsis. Mechanistic studies are needed to further understand the alterations in release and response of adiponectin in sepsis and its role in the development of lung injury.

## Key messages

- Low plasma adiponectin levels at the time of ICU admission were not associated with ARDS in human patients with severe sepsis and septic shock
- The association between plasma adiponectin levels and ARDS is independent of BMI
- Further research is needed to determine whether circulating adiponectin has a pathogenic role in the development of ARDS

## Abbreviations

APACHE: Acute Physiology and Chronic Health Evaluation
ARDS: acute respiratory distress syndrome
BMI: body mass index
FACTT: Fluid and Catheters Treatment Trial
ICU: intensive care unit
LPS: lipopolysaccharide
MESSI: Molecular Epidemiology of Severe Sepsis in the ICU
OR: odds ratio
